# The E3 ubiquitin ligase Smurf2 regulates PARP1 stability to alleviate oxidative stress‐induced injury in human umbilical vein endothelial cells

**DOI:** 10.1111/jcmm.15121

**Published:** 2020-03-13

**Authors:** Hao Qian, Naijin Zhang, Boquan Wu, Shaojun Wu, Shilong You, Ying Zhang, Yingxian Sun

**Affiliations:** ^1^ Department of Cardiology the First Hospital of China Medical University Shenyang China

**Keywords:** apoptosis, Smurf2, ubiquitination

## Abstract

Oxidative stress injury is involved in many cardiovascular diseases, like hypertension and myocardial infarction. Ubiquitination is a ubiquitous protein post‐translational modification that controls a wide range of biological functions and plays a crucial role in maintaining the homeostasis of cells in physiology and disease. Many studies have shown that oxidative stress damage is inextricably linked to ubiquitination. We demonstrate that Smurf2, an E3 ubiquitinated ligase, is involved in HUVEC apoptosis induced by oxidative stress to alleviate H_2_O_2_‐induced reactive oxygen species (ROS) production and the apoptosis of human umbilical vein endothelial cells (HUVECs). At the same time, we found that Smurf2 can bind the poly(ADP‐ribose) polymerase‐1(PARP1), and the interaction is enhanced under the stimulation of oxidative stress. We further study and prove that Smurf2 can promote PARP1 ubiquitination and degradation. Collectively, we demonstrate Smurf2 degradation of overactivated PARP1 by ubiquitin‐proteasome pathway to protect HUVEC and alleviate oxidative stress injury.

## 1 INTRODUCTION

In the coming decades, the number of people over the age of 65 is expected to increase dramatically.^1^ The ageing of the population will gradually increase the incidence of cardiovascular diseases,^2^, ^3^ which seriously affects human health. In many physiological activities of the cardiovascular system, ROS are important signalling molecules that maintain the homeostasis of the internal environment of the cardiovascular system.^4^ A significant increase in ROS levels is closely related to the occurrence and development of cardiovascular diseases including heart failure,^5^ atherosclerosis,^6^ hypertension^7^ and ischaemic heart disease.^8^ Furthermore, these diseases are based on endothelial dysfunction caused by oxidative stress (including inflammation, fibrosis and apoptosis).^9^, ^10^ Therefore, the causes and mechanisms of endothelial dysfunction caused by oxidative stress have always been the main focus of cardiovascular research.

PARP1 is a highly conserved protein approximately 116 kD in size. Previous literature has reported that PARP1, a cleavage substrate of caspase3, plays an irreplaceable role in apoptosis.^11^ In the cardiovascular system, the activation of PARP1 is often detrimental. In hypertension and hyperglycaemia, activated PARP1 can cause vascular endothelial damage.^12^ Additionally, decreasing the activity of PARP1 in atherosclerosis can attenuate the development of atherosclerotic plaques, enhance the stability of plaques and promote pre‐established atherosclerotic plaque regression.^13^ In animal models, the pharmacological inhibition of PARP1 or its gene deletion can reduce tissue damage associated with myocardial ischaemia‐reperfusion.^14^, ^15^ In addition, PARP1 inhibitors can also reverse the pattern of cell death from necrosis to apoptosis. However, the regulatory mechanism of PARP1 in endothelial injury remains unclear.

Smurf2, a C2‐WW‐HECT‐domain E3 ubiquitin ligase belonging to the NEDD4 subfamily of HECT‐type E3 ligases, is similar to other NEDD4 family members (9 in total).^16^ Smurf2 participates in many cellular physiological processes through the ubiquitin‐proteasome pathway. Smurf2 also affects embryonic development, cell polarity, cell migration and bone homeostasis.^17^ In the past 10 years, the ubiquitin‐proteasome system has been widely studied in the cardiovascular field, including its role in atherosclerosis, familial cardiac proteinopathies, idiopathic dilated cardiomyopathies and myocardial ischaemia.^18^ However, the role and mechanism of Smurf2 in endothelial cell injury induced by oxidative stress remain unclear.

Here, we describe the regulation of PARP1 by Smurf2 in HUVECs injured by H_2_O_2._ Smurf2 was significantly increased following H_2_O_2_‐induced HUVEC injury. Knockdown of Smurf2 significantly increased HUVEC apoptosis, and overexpression of Smurf2 rescued this effect. Based on this mechanism, coimmunoprecipitation confirmed that Smurf2 is a new protein that interacts with and degrades PARP1 through the ubiquitin‐proteasome system, which alleviates endothelial cell apoptosis under oxidative stress.

## 2 MATERIALS AND METHODS

### 2.1 Plasmids

Flag‐Smurf2 was constructed by Obio Technology Corp., and Myc‐PARP1 and HA‐UB were purchased from GeneChem. Plasmids of Smurf2 and PARP1 functional domains were purchased from GeneChem. Smurf2 (C716G) amplified by PCR. The sequences of the primers used were as follows: Fwd:5′‐CGAAAGCCCACACTGGCTTCAATCGAATAG‐3′, Rev:5′‐CTATTCGATTGAAGCCAGTGTGGGCTTTCG‐3′. PCR was performed with the following parameters: 94°C for 5 minutes; 25 cycles of 94°C for 30 seconds, 55°C for 1 minute and 72°C for 6 minutes; and finally 72°C for 10 minutes. Mutation was confirmed by DNA sequencing. All plasmids were transfected with Lipofectamine 3000 from Invitrogen with Opti‐MEM from Gibco for 48 hours. The sh‐Smurf2 plasmid was purchased from GeneChem. The sequences of the three sh Smurf2 sequences were as follows: 5′‐CTGCAGTCGTTTATTTGAT‐3′, 5′‐GCTGCTTTGTTGATGAGAA‐3′, 5′‐CACTCCAATTAGTGGAACA‐3′. The efficiency of Smurf2 knockdown was confirmed by Western blot analysis.

### 2.2 SiRNA interference

Smurf2 siRNA was provided by RiboBio. jetPRIME transfection reagent from Polyplus was used as the medium. HUVECs at 60% confluence were transfected with control siRNA and Smurf2 siRNAs (1 stB0001237A, 2 stB0001237B and 3 stB0001237C) for 72 hours. After 12 hours of siRNA transfection, the medium was changed. The cells were then stimulated with H_2_O_2_ stimulation for the appropriate length of time. The efficiency of Smurf2 knockdown was confirmed by Western blot analysis.

### 2.3 Antibodies and reagents

The following antibodies were used: anti‐caspase3 (D3R6Y, mAb #14220, Cell Signaling Technology, WB: 1:1000), anti‐Smurf2 (D8B8, mAb #12024, Cell Signaling Technology, WB: 1:1000, IF: 1:50), anti‐PARP1 (46D11, mAb #9532, Cell Signaling Technology, WB: 1:1000, IF: 1:200), anti‐MY (9B11, mAb #2276, Cell Signaling Technology, WB: 1:1000), anti‐Flag(9A3, mAb #8146, Cell Signaling Technology, WB: 1:1000, IF: 1:50), anti‐HA (6E2, mAb #2367, Cell Signaling Technology, WB: 1:1000) and anti‐β‐tubulin (10094‐1‐AP, Proteintech, WB: 1:1000). Protein A/G magnetic beads were acquired from Biotool. Cycloheximide (A8244) and MG132 (A2585) were purchased from ApexBio. Heclin (5433) was purchased from Tocris a bio‐techne brand.

### 2.4 Cell culture and treatment

Human umbilical vein endothelial cells, a human umbilical vein endothelial cell line, were cultured in F‐12K medium with 10% FBS (HyClone), 0.1 mg/mL heparin and 5 mL of endothelial cell growth supplement. 293T cells (a human embryonic kidney cell line) were cultured in high‐glucose DMEM with 10% FBS (HyClone). All cells were maintained at 37°C and 5% CO_2_. Culture media were replaced every day. When the cells were approaching confluence, they were passaged with trypsin (0.25%) at a ratio of 1:3. H_2_O_2_ has been widely used in vitro models to induce injury and the apoptosis of human vascular endothelial cells.

### 2.5 Cell viability assay

A Cell Counting Kit‐8 assay (Dojindo) was used to analyse HUVEC viability as follows. A total of 10^4^ cells/well were seeded into 96‐well plates (NEST Biotechnology) in F‐12K complete culture medium. Different groups of HUVECs were transfected for 48 or 72 hours, stimulated with PBS or H_2_O_2_ (250, 500, 750 μmol/L) for 12 hours and incubated with 100 μL of CCK‐8 solution per well for 2 hours, following which cell viability was measured at 450 nm by scanning with a Bio‐Rad microplate reader (Model 680; Bio‐Rad Laboratories, Inc). Finally, statistical software was used to calculate cell survival and viability.

### 2.6 Flow cytometry and annexin‐FITC/PI staining

Annexin V‐fluorescein isothiocyanate (FITC) and propidium iodide (PI) staining were used to detect cell apoptosis according to the manufacturer's instructions, followed by flow cytometry. The different groups of cells were transfected for 48 or 72 hours and stimulated with H_2_O_2_ at the corresponding time. The cells were collected with 0.25% trypsin without EDTA. Next, the cells were incubated in 700 μL of binding buffer with 4 μL of annexin V and 2 μL of a PI solution for 15 minutes at RT in the dark. Finally, fluorescence was measured with the FL‐1 and FL‐2 channels of A FACSCalibur flow cytometer.

### 2.7 ROS assay

An ROS Assay Kit used to detect intracellular reactive oxygen species (Beyotime Biotechnology). HUVECs were exposed to H_2_O_2_ (0, 250, 500, 750 μmol/L) or PBS for 12 hours. Other groups were transfected for 48 hours or 72 hours and exposed to H_2_O_2_ (500 μmol/L) or PBS for 12 hours. After washing with PBS three times, the cells were suspended in 500 μL of serum‐free medium with 2,7‐DCFH‐DA (10 mmol/L) for 60 minutes and then maintained at 37°C and 5% CO2 in the dark. Then, the cells were washed with PBS three times. The cells were excited at 488 nm and imaged at 525 nm by fluorescence microscopy.

### 2.8 Coimmunoprecipitation and Western blot assays

The cells were washed with PBS three times and lysed with cell lysis buffer (50 mmol/L Tris, 137 mmol/L NaCl, 1 mmol/L MEDTA, 10 mmol/L NaF, 0.1 mmol/L Na3VO4, 1% NP‐40, 1 mmol/L DTT, 10% glycerol, pH 7.8 and 100 × protease inhibitor, (Roche)). After centrifugation (4°C, 17 000 *g*, 15 minutes), the cell lysates were incubated with specific antibodies and 30 μL of magnetic beads at 4°C for 12 hours. Then, the bound complexes were washed with cell lysis buffer and subjected to SDS‐PAGE. Protein samples were separated by 8% or 12% SDS‐polyacrylamide gels and transferred to PVDF membranes (Millipore USA). After blocking with Tris‐buffered saline containing Tween (TBS‐T) with 5% bovine serum albumin (BSA) at RT for 1 hour, the membranes were incubated with 1% BSA, which diluted the corresponding antibody, at 4°C overnight.

### 2.9 Statistical analysis

All data were processed by SPSS version 22.0 software and GraphPad Prism 6 software. Data are mean ± standard deviation (SD). Homogeneity of variance was evaluated by the F test (group pair). The Shapiro‐Wilk test was performed for assessing data normality. Non‐parametric tests were employed to assess data of two groups. *P* values were adjusted for multiple comparisons when applicable. *P* < .05 was considered statistically significant.

## 3 RESULTS

### 3.1 Smurf2 participates in HUVEC injury induced by H_2_O_2_

**Figure 1:**
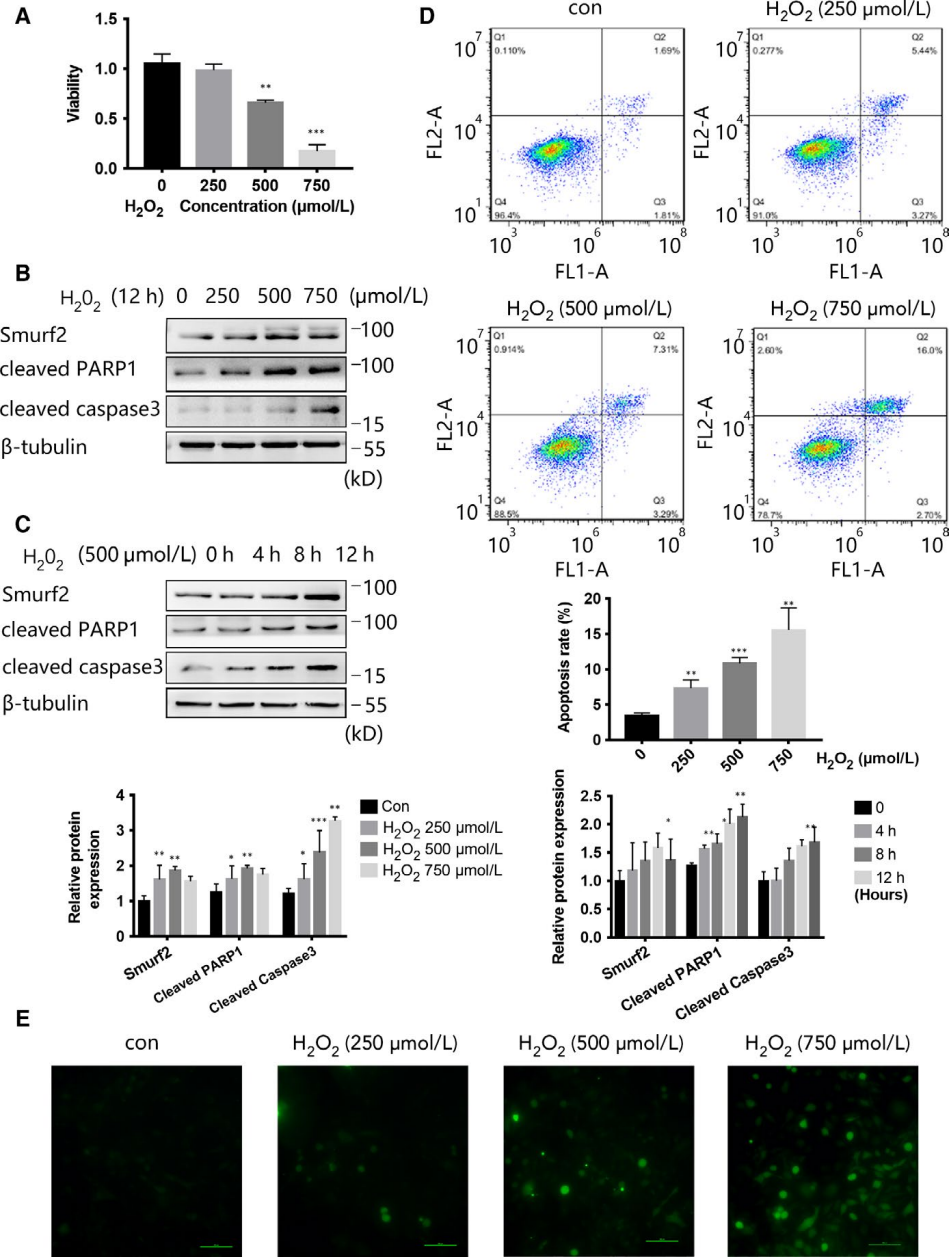
H_2_O_2_ increased the expression of Smurf2 and apoptosis‐related proteins and decreases the viability of HUVEC. A, HUVEC was treated with H_2_O_2_ at the concentrations of 0, 250, 500 and 750 μmol/L for 12 h, and cell viability was determined by CCK8 assay. B, HUVEC was treated with H_2_O_2_ at the concentrations of 0, 250, 500 and 750 μmol/L for 12 h, and the expression of Smurf2, cleaved PARP1 and cleaved caspase3 was analysed by Western blot. β‐ tubulin was used as a loading control. ***P < .001, **P < .01 and *P < .05. Data were expressed as the mean ± SD of triplicate experiments. C, HUVEC was treated with H_2_O_2_ for 0, 4, 8 and 12 h at a concentration of 500 μmol/L, and the expression of Smurf2, cleaved PARP1 and cleaved caspase3 was analysed by Western blot. β‐ tubulin was used as a loading control. ***P < .001, **P < .01 and *P < .05. Data were expressed as the mean ± SD of triplicate experiments. D, Flow cytometry annexin‐FITC/PI results indicate the apoptosis rate of HUVEC, and HUVEC was treated with H_2_O_2_ at the concentrations of 0, 250, 500 and 750 μmol/L for 12 h. E, HUVEC was treated with H_2_O_2_ at the concentrations of 0, 250, 500 and 750 μmol/L for 12 h. Staining with 2,7‐DCFH‐DA, the results showed the accumulation of reactive oxygen species by using fluorescence microscopy at 488 nm and imaged at 525 nm

Previous studies have not assessed the expression of Smurf2 following oxidative stress‐induced HUVEC injury. We stimulated HUVECs with H_2_O_2_ at the concentrations of 0, 250, 500 and 750 μmol/L for 12 hours and H_2_O_2_ at the concentrations of 500 μmol/L for 0, 4, 8 and 12 hours before observing the expression of Smurf2 (Figure 1B,C). With increasing H_2_O_2_ treatment time and concentration, the expression of Smurf2, cleaved PARP1 and cleaved caspase3 was obviously up‐regulated. And under the above stimulation (H_2_O_2_ at the concentrations of 0, 250, 500 and 750 μmol/L for 12 hours), HUVEC viability was also significantly reduced (Figure 1A). And with H_2_O_2_ at the concentrations of 0, 250, 500 and 750 μmol/L for 12 hours, flow cytometry following annexin‐FITC/PI staining showed that apoptosis rate increased (Figure 1D). Through ROS Assay Kit to detect intracellular ROS, we stimulated HUVECs with different concentrations of H_2_O_2_ (0, 250, 500 and 750 μmol/L, 12 hours). Firstly, we observed the accumulation of ROS (Figure 1E), and we suggested that H_2_O_2_ treatment can increase ROS in the cells. Therefore, we speculated that Smurf2 is involved in H_2_O_2_‐induced HUVEC injury.

### 3.2 Smurf2 can alleviate HUVEC damage caused by H_2_O_2_‐induced oxidative stress

**Figure 2:**
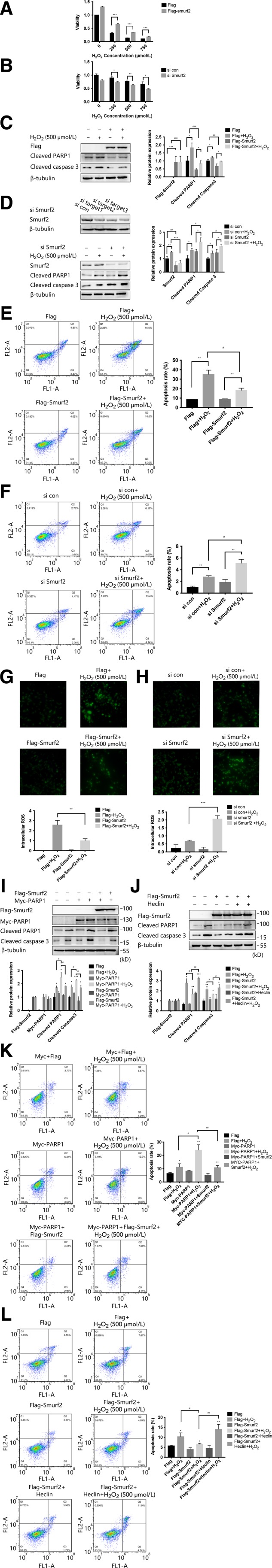
Smurf2 can alleviate HUVEC damage caused by H_2_O_2_‐induced oxidative stress. A, Transfection of HUVEC with Flag‐Smurf2, HUVEC was treated with H_2_O_2_ at the concentrations of 0, 250, 500 and 750 μmol/L for 12 h and cell viability was determined by CCK8 assay. B, Knockdown Smurf2 with control siRNA and Smurf2 siRNA target sequences in HUVEC, HUVEC was treated with H_2_O_2_ at the concentrations of 0, 250, 500 and 750 μmol/L for 12 h and cell viability was determined by CCK8 assay. C, Transfection of HUVEC with Flag‐Smurf2, HUVEC was treated with H_2_O_2_ at the concentrations of 500 μmol/L, 12 h and the expression of Flag, cleaved PARP1 and cleaved caspase3 was analysed by Western blot. β‐ tubulin was used as a loading control. ***P < .001, **P < .01 and *P < .05. Data were expressed as the mean ± SD (^###^ *P* < .001, ^##^ *P* < .01 and ^#^ *P* < .05, non‐parametric tests). D, Knockdown Smurf2 with control siRNA and Smurf2 siRNA in HUVEC, HUVEC was treated with H_2_O_2_ at the concentrations of 500 μmol/L, 12 h and the expression of Smurf2, cleaved PARP1 and cleaved caspase3 was analysed by Western blot. β‐tubulin was used as a loading control. ***P < .001, **P < .01 and *P < .05. Data were expressed as the mean ± SD (^###^ *P* < .001, ^##^ *P* < .01, ^#^ *P* < .05 and non‐parametric tests). E, Flow cytometry annexin‐FITC/PI results indicate the apoptosis rate of HUVEC after overexpression of Flag‐Smurf2, under the oxidative stress (H_2_O_2_ 500 μmol/L, 12 h). F, Flow cytometry annexin‐FITC/PI results indicate the apoptosis rate of HUVEC after knockdown Smurf2, under the oxidative stress (H_2_O_2_ 500 μmol/L, 12 h). G, Transfection of HUVEC with Flag‐Smurf2 and treated with H_2_O_2_ at the concentrations of 500, μmol/L for 12 h. Staining with 2,7‐DCFH‐DA, the results showed the accumulation of reactive oxygen species by using fluorescence microscopy at 488 nm and imaged at 525 nm. H, Knockdown of Smurf2 of HUVEC and treated with H_2_O_2_ at the concentrations of 500 μmol/L, 12 h. Staining with 2,7‐DCFH‐DA, the results showed the accumulation of reactive oxygen species by using fluorescence microscopy at 488 nm, and imaged at 525 nm. I, Transfection of HUVEC with Myc‐PARP1 and Flag‐Smurf2, HUVEC was treated with H_2_O_2_ at the concentrations of 500 μmol/L, 12 h and the expression of Flag, Myc, cleaved PARP1 and cleaved caspase3 was analysed by Western blot. β‐tubulin was used as a loading control. ***P < .001, **P < .01 and *P < .05. Data were expressed as the mean ± SD (^###^ *P* < .001, ^##^ *P* < .01, ^#^ *P* < .05 and non‐parametric tests). J, Transfection of HUVEC with Flag‐Smurf2, HUVEC was treated with H_2_O_2_ at the concentrations of 500 μmol/L, 12 h and Heclin 25 μmol/L, 2 h. the expression of Flag, cleaved PARP1 and cleaved caspase3 was analysed by Western blot. β‐tubulin was used as a loading control. ***P < .001, **P < .01 and *P < .05. Data were expressed as the mean ± SD (^###^ *P* < .001, ^##^ *P* < .01, ^#^ *P* < .05 and non‐parametric tests). K, Flow cytometry annexin‐FITC/PI results indicate the apoptosis rate of HUVEC after overexpression of Flag‐Smurf2 and Myc‐PARP1, under the oxidative stress (H_2_O_2_ 500 μmol/L, 12 h). L, Flow cytometry annexin‐FITC/PI results indicate the apoptosis rate of HUVEC after overexpression of Flag‐Smurf2 under the oxidative stress (H_2_O_2_ 500 μmol/L, 12 h) and Heclin (25 μmol/L, 2 h)

To further explore the role of Smurf2 in H_2_O_2_‐induced HUVEC injury, HUVECs were transfected with empty vector and a Flag‐Smurf2 plasmid and then stimulated with H_2_O_2_ (500 μmol/L, 12 hours). We found that the overexpression of Smurf2 significantly reduced the expression of cleaved PARP1 and cleaved caspase3 and decreased HUVEC apoptosis (Figure 2C). A HUVEC viability assay (H_2_O_2_ 0, 250, 500 and 750 μmol/L, 12 hours) and flow cytometry following annexin‐FITC/PI staining (H_2_O_2_ 500 μmol/L, 12 hours) showed that the overexpression of Smurf2 alleviated apoptosis (Figure 2A,E) Therefore, these results indicated that the overexpression of Smurf2 can attenuate HUVEC apoptosis caused by oxidative stress to a certain extent.

We then identified the most efficacious Smurf2 siRNA fragment from three fragments. We transfected HUVECs with control siRNA and siRNA fragment 3 and then stimulated the HUVECs with H_2_O_2_ (500 μmol/L, 12 hours). The expression of cleaved PARP1 and cleaved caspase3 in Smurf2 knockdown cells was significantly higher than that in the negative control group, and HUVEC injury was aggravated (Figure 2D). The results of a HUVEC viability assay (H_2_O_2_ 0, 250, 500 and 750 μmol/L, 12 hours) and flow cytometry after annexin‐FITC/PI staining (H_2_O_2_ 500 μmol/L, 12 hours) also showed that Smurf2 knockdown increased apoptosis (Figure 2B,F). Therefore, the knockdown of Smurf2 increases HUVECs apoptosis induced by oxidative stress. Through the above experiments, we determined that the overexpression of Smurf2 can reduce oxidative stress damage to HUVECs, thus protecting HUVECs. Conversely, the knockdown of Smurf2 can increase the toxic effect of oxidative stress on HUVECs.

To clarify the role of Smurf2 in oxidative stress, we detected ROS. We overexpressed Flag‐Smurf2 and stimulated with H_2_O_2_ (500 μmol/L, 12 hours) to observe intracellular ROS accumulation. We knocked down Smurf2 and stimulate with H_2_O_2_(500 μmol/L, 12 hours) to observe intracellular ROS accumulation. We found that overexpression of Smurf2 can inhibit the production of ROS (Figure 2G), and Smurf2 knockdown increased the accumulation of ROS (Figure 2H). Through the above experiments, we confirmed that Smurf2 can alleviate the accumulation of intracellular ROS induced by H_2_O_2_. Therefore, Smurf2 acts as a protective factor in H_2_O_2_‐induced HUVEC injury.

We overexpressed Myc‐PARP1 and Flag‐Smurf2 together in HUVEC, we have observed that overexpression of Myc‐PARP1 can aggravate oxidative stress injury of H_2_O_2_ (500 μmol/L, 12 hours), and overexpression of Flag‐Smurf2 on the basis of overexpression of Myc‐PARP1 can rescue the damage to some extent (Figure 2I). At the same time, flow cytometry following annexin‐FITC/PI staining (H_2_O_2_ 500 μmol/L, 12 hours) results were the same as before (Figure 2K).

We bought the Smurf2 inhibitor Heclin, then overexpressed Flag‐Smurf2 and gave Smurf2 inhibitor Heclin (25 μmol/L, 2 hours) in HUVEC. Under the stimulation of oxidative stress (H_2_O_2_ 500 μmol/L, 12 hours), we confirmed that Smurf2 lost its ability to protect HUVEC from oxidative stress injury (Figure 2J) because Heclin blocked the activity of Smurf2. The flow cytometry following annexin‐FITC/PI staining (H_2_O_2_ 500 μmol/L, 12 hours, Heclin 25 μmol/L, 2 hours) results also confirm this conclusion (Figure 2L). We conclude from the above experiments: overexpression of Myc‐PARP1 can aggravate oxidative stress injury of HUVEC, and Smurf2 can rescue this phenomenon. Then, the inhibition of Smurf2 activity makes Smurf2 lose the ability to alleviate the oxidative stress injury of HUVEC.

### 3.3 Smurf2 interacts with PARP1, and this interaction is enhanced after oxidative stress stimulation

**Figure 3:**
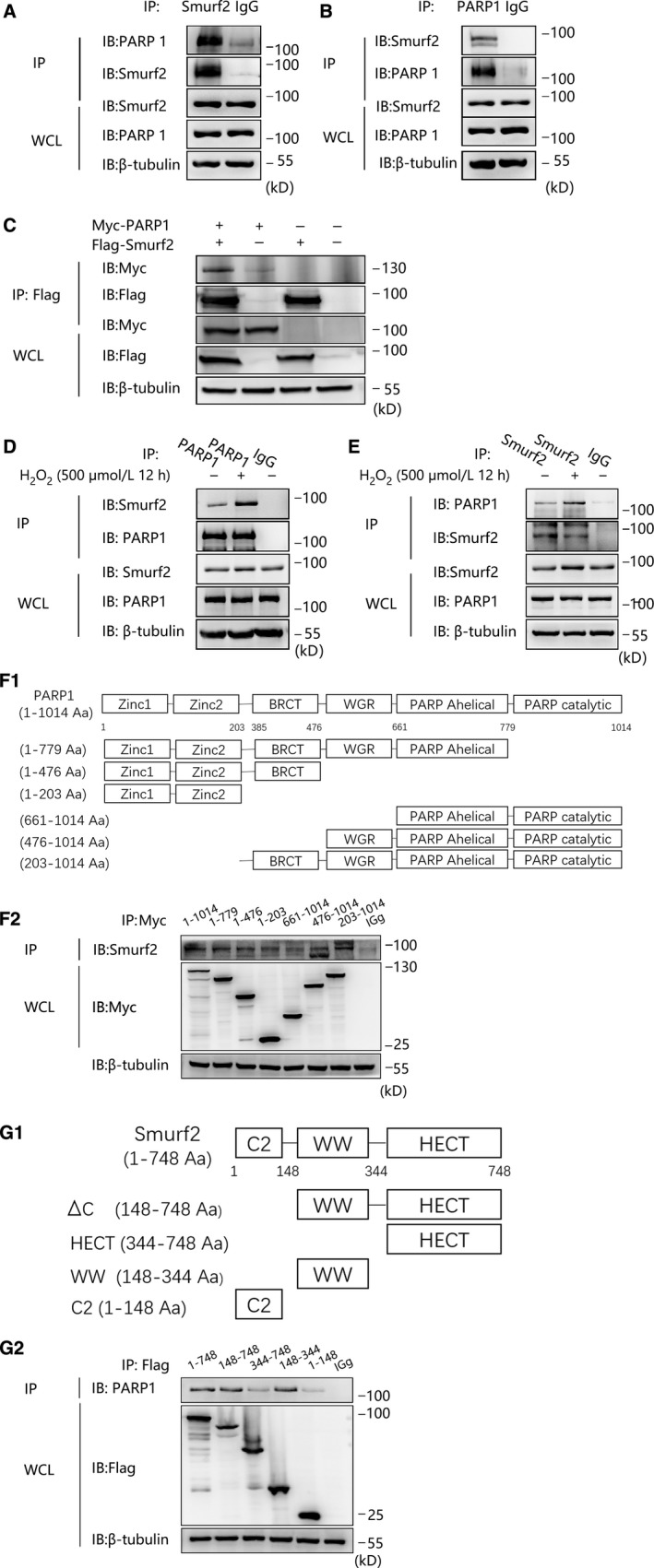
The interaction between PARP1 and Smurf2. A and B, Coimmunoprecipitation (co‐IP) and Western blotting (IP‐western) using anti‐Smurf2 or anti‐PARP1 antibody or negative control IgG and Protein A/G immunoprecipitation magnetic beads followed by anti‐PARP1 or anti‐Smurf2 Western blot were used to verify endogenous interaction between Smurf2 and PARP1. C, Transfection of 293T cells with Flag‐Smurf2 together with Myc‐PARP1 for 48 h, Anti‐Flag antibody and Protein A/G immunoprecipitation magnetic beads followed by anti‐Myc Western blot to verify the exogenous interaction between Flag‐Smurf2 and Myc‐PARP1. D and E, The endogenous interaction between Smurf2 and PARP1 was enhanced by treatment of 500 μmol/L H_2_O_2_ for 12 h in HUVEC. F and G, Indicated truncates of PARP1 and Smurf2 were constructed according to their functional domains. Transfection of 293T with the indicated truncates of Myc‐PARP1 or Flag‐Smurf2. Coimmunoprecipitation (co‐IP) and Western blotting (IP‐western) using anti‐Myc or anti‐Flag antibody or negative control IgG and Protein A/G immunoprecipitation magnetic beads followed by anti‐Smurf2 or anti‐PARP1 Western blot were used to verify the interaction between Smurf2 and PARP1

The previous results demonstrated that Smurf2 participates in and alleviates H_2_O_2_‐induced HUVEC injury, thereby playing a positive role in protecting HUVECs. We then determined the mechanism by which Smurf2 attenuates oxidative damage. We confirmed by endogenous coimmunoprecipitation that Smurf2 is a novel protein that interacts with PARP1 (Figure 3A,B), We overexpressed Flag‐Smurf2 and Myc‐PARP1 for 48 hours in 293T cells, through the exogenous coimmunoprecipitation to verify the exogenous interaction between Flag‐Smurf2 and Myc‐PARP1 (Figure 3C). And that Smurf2 and PARP1 enhance each other after stimulation with H_2_O_2_ (500 μmol/L, 12 hours) (Figure 3D,E).

To map the binding region between Smurf2 and PARP1. We indicated truncates of PARP1 and Smurf2 were constructed according to their functional domains (Figure 3F1,G1). Six truncated plasmids of PARP1 were produced. ΔPARP catalytic domain plasmids, containing Zinc finger and BRCT domain plasmids, Zinc finger domain plasmids and the domains of the remaining three plasmids, are complementary to the domains of previous three plasmids. We overexpressed Myc‐PARP1‐truncated plasmid in 293T cells for immunocoprecipitation, confirming that BRCT domain is the main domain combined with Smurf2 (Figure 3F2).

Next, we constructed the truncated plasmid of Smurf2 ΔC domain, HECT domain, WW domain and C2 domain. We overexpressed the Flag‐Smurf2‐truncated plasmids for immunocoprecipitation, and WW domain alone can interact with PARP1, indicating that WW domains in Smurf2 mediate its interaction with PARP1 (Figure 3G2). Consequently, Smurf2 may attenuate HUVEC damage by interacting with activated PARP1.

### 3.4 Smurf2 can affect the stability of PARP1

**Figure 4:**
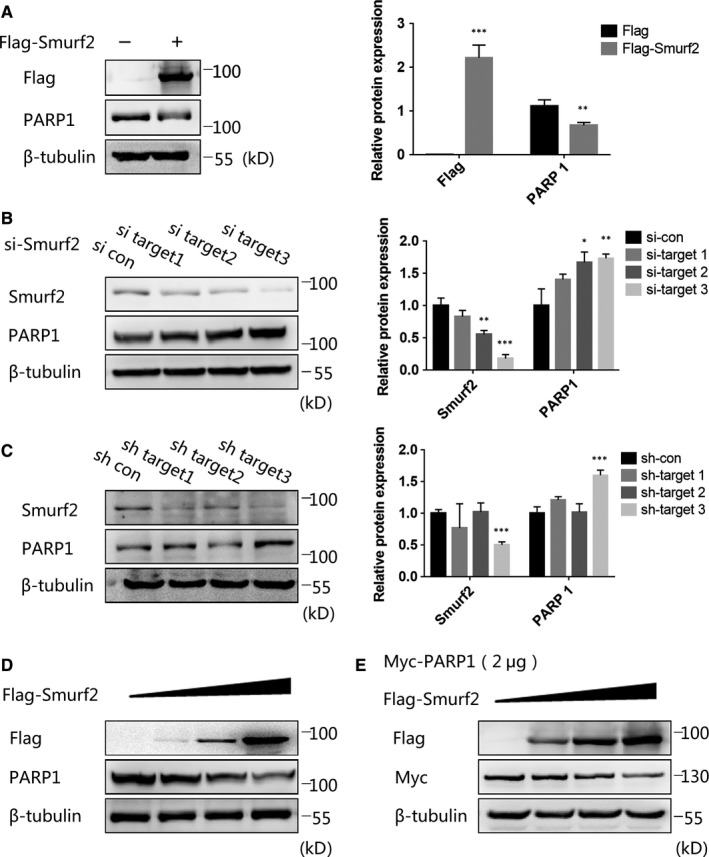
Smurf2 can affect the stability of PARP1. A, Transfection of 293T cells with Flag‐Smurf2 for 48 h, overexpression of Smurf2 can reduce the expression of endogenous PARP1 by Western blot. β‐tubulin was used as a loading control. ***P < .001, **P < .01 and *P < .05. Data were expressed as the mean ± SD of triplicate experiments. B, Knockdown Smurf2 with control siRNA and Smurf2 siRNA target sequences 1, 2 and 3, the expression of PARP1 is positively correlated with the knockdown efficiency of Smurf2 by Western blot. β‐tubulin was used as a loading control. ***P < .001, **P < .01 and *P < .05. Data were expressed as the mean ± SD of triplicate experiments. C, Construction of Smurf2 knockdown stable cell line in HUVEC. The expression of PARP1 is positively correlated with the knockdown efficiency of Smurf2 by Western blot. β‐tubulin was used as a loading control. ***P < .001, **P < .01 and *P < .05. Data were expressed as the mean ± SD of triplicate experiments. D, Gradient overexpression of Flag‐Smurf2 in 293T cells, the expression of PARP1 was negatively correlated with the transfection amount of Flag‐Smurf2 by Western blot. β‐tubulin was used as a loading control. E, Gradient overexpression of Flag‐Smurf2 in 293T cells together with Myc‐PARP1 (2 μg), the expression of Myc‐PARP1 was negatively correlated with the transfection amount of Flag‐Smurf2 by Western blot. β‐tubulin was used as a loading control

The overexpression of Flag‐Smurf2 affected the expression of PARP1 (Figure 4A), which indicates that Smurf2 can regulate the stability of PARP1. The expression of PARP1 was significantly up‐regulated in cells transfected with three siRNA fragments against Smurf2 (Figure 4B). Similar results were observed in a cell line in which Smurf2 had been stably silenced (Figure 4C). Then, we found that both endogenous and exogenous PARP1 expression depended on and were negatively correlated with the expression of Flag‐Smurf2 (Figure 4D,E). Therefore, Smurf2 negatively regulate PARP1.

### 3.5 Smurf2 mediates PARP1 degradation through the proteasome pathway

Smurf2 is an important E3 ubiquitin ligase. The increased expression of Smurf2 significantly reduced the expression of PARP1, whereas Smurf2 knockdown increased the expression of PARP1. These results strongly suggested PARP1 as a new substrate of Smurf2, indicating that Smurf2 may degrade PARP1 in some way and this degradation likely occurs via the proteasome pathway.

**Figure 5:**
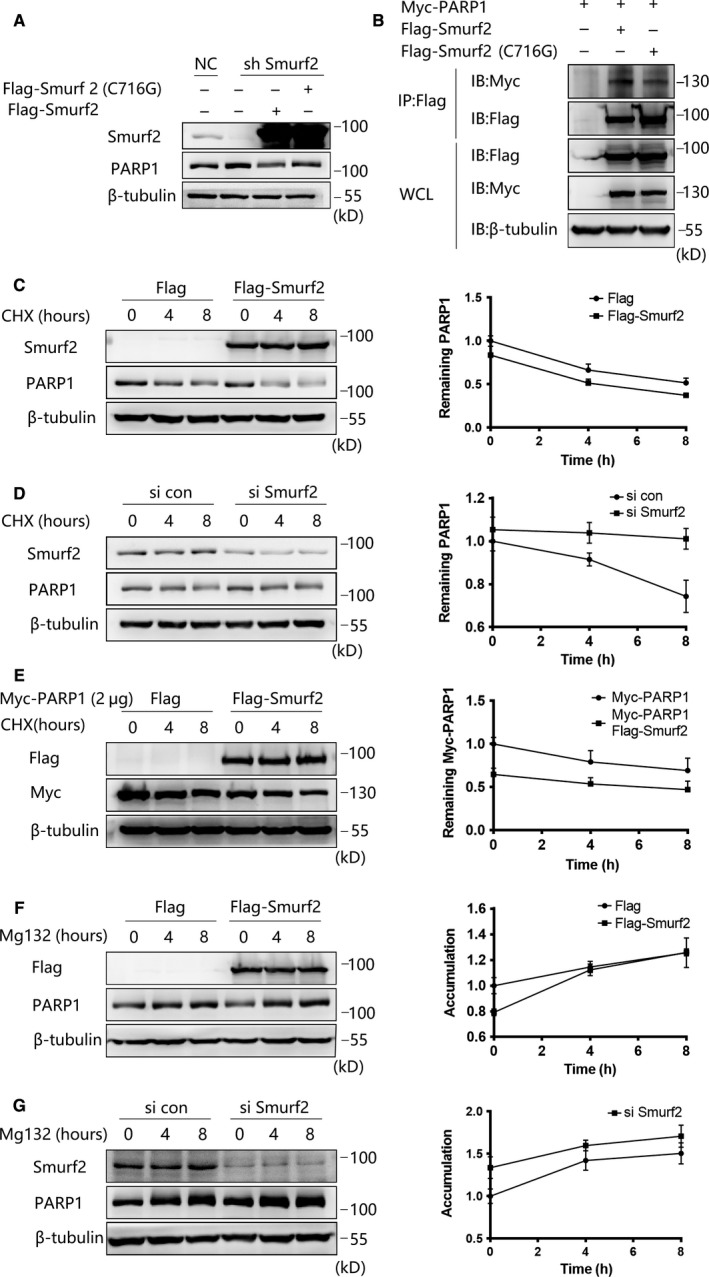
Smurf2 mediates PARP1 degradation through proteasome pathway. A, Overexpression of Flag‐Smurf2 and Flag‐Smurf2 (C716G) in HUVEC stably knocking down of Smurf2, Flag‐Smurf2 (C716G) lost the negative regulation of PARP1 by Western blot. β‐tubulin was used as a loading control. B, Transfection of 293T cells with Flag‐Smurf2 and Flag‐Smurf2 (C716G) in 293T cells together with Myc‐PARP1 for 48 h, anti‐Flag antibody and Protein A/G immunoprecipitation magnetic beads followed by anti‐Myc Western blot, the interaction between Flag‐Smurf2 (C716G) and Myc‐PARP1 is weakened. C, Transfection of 293T cells with Flag‐Smurf2 and cells were treated with CHX at 100 μmol/L or DMSO for the indicated times. The half‐life of PARP1 was measured by Western blot. β‐tubulin was used as a loading control, and each point is represented as the mean ± SD of triplicate experiments. D, Knockdown Smurf2 with control siRNA and Smurf2 siRNA in 293T cells were treated with CHX at 100 μmol/L or DMSO for the indicated times. The half‐life of PARP1 was measured by Western blot. β‐tubulin was used as a loading control, and each point is represented as the mean ± SD of triplicate experiments. E, Transfection of Flag‐Smurf2 in 293T cells together with Myc‐PARP1, the cells were treated with CHX at 100 μmol/L or DMSO for the indicated times. The half‐life of Myc‐PARP1 was measured by Western blot. β‐tubulin was used as a loading control, and each point is represented as the mean ± SD of triplicate experiments. F, Transfection of Flag‐Smurf2 in 293T cells and cells were treated with MG132 at 25 μmol/L or DMSO for the indicated times. The accumulation of PARP1 was measured by Western blot. β‐tubulin was used as a loading control, and each point is represented as the mean ± SD of triplicate experiments. G, Knockdown Smurf2 with control siRNA and Smurf2 siRNA in 293T cells were treated with MG132 at 25 μmol/L or DMSO for the indicated times. The accumulation of PARP1 was measured by Western blot. β‐tubulin was used as a loading control and each point is represented as the mean ± SD of triplicate experiments

According to the literature, we constructed a plasmid in which a functional site had been deactivated called Flag‐smurf2 (C716G). We overexpressed Flag‐Smurf2 and Flag‐Smurf2 (C716G) in cells in which Smurf2 had been stably knocked down. The expression of endogenous PARP1 in Smurf2 mutant cells was higher than that in cells transfected with Flag‐Smurf2 (Figure 5A). Furthermore, the interaction between Smurf2 (C716G) and PARP1 was weakened, as shown by coimmunoprecipitation experiments (Figure 5B). These results suggest that the negative regulation of PARP1 by Smurf2 is related to the ubiquitination of PARP1 by Smurf2.

To further validate our hypothesis, we overexpressed Flag‐Smurf2 and treated the resulting cells with cycloheximide (CHX); the half‐life of endogenous PARP1 in these CHX‐treated cells was shorter than that in the empty vector group (Figure 5C). Next, we treated Smurf2 knockdown cells with CHX and found that the knockdown of Smurf2 increased the shortened half‐life of endogenous PARP1 (Figure 5D). The same experimental results were obtained in CHX‐treated cells overexpressing Myc‐PARP1 and Flag‐Smurf2 (Figure 5E). The same experimental results were obtained in CHX‐treated cells overexpressing Myc‐PARP1 and Flag‐Smurf2 (Figure 5E).

To further explore how Smurf2 affects the stability of PARP1, we transfected cells with empty vector Flag‐Smurf2 and then administered the proteasome pathway inhibitor MG132. We found that MG132 could block the Smurf2‐mediated negative regulation of PARP1 and that endogenous PARP1 accumulation was more pronounced (Figure 5F). This accumulation of PARP1 was further amplified when MG132 was administered to Smurf2 knockdown cells (Figure 5G). These results confirmed that Smurf2 can degrade PARP1 through the proteasome pathway.

### 3.6 Smurf2 mediates the polyubiquitination of PARP1

**Figure 6:**
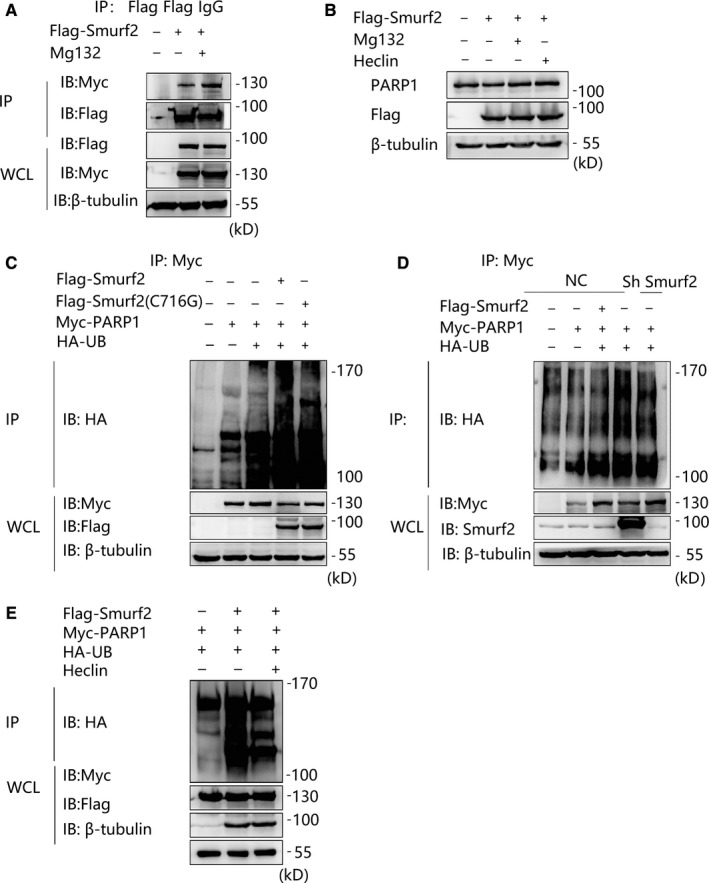
Smurf2 degrade PARP1 by polyubiquitination. A, Transfection of Flag‐Smurf2 in 293T cells together with Myc‐PARP1, the cells were treated with MG132 at 25 μmol/L or DMSO for 8 h. Anti‐Myc antibody and Protein A/G immunoprecipitation magnetic beads followed by anti‐Flag by Western blot. B, Transfection of Flag‐Smurf2 in 293T cells and the cells were treated with MG132 at 25 μmol/L or DMSO for 8 h, and Heclin 25 μmol/L, 2 h. The accumulation of PARP1 was measured by Western blot. β‐tubulin was used as a loading control. C, Overexpression of Flag‐Smurf2 in 293T cells together with Myc‐PARP1 and HA‐UB, anti‐Myc antibody and Protein A/G immunoprecipitation magnetic beads followed by anti‐HA Western blot a to verify the ubiquitination of Myc‐PARP1. D, Transfection of Flag‐Smurf2, Myc‐PARP1 and HA‐UB in HUVEC stably knocking down of Smurf2, anti‐Myc antibody and Protein A/G immunoprecipitation magnetic beads followed by anti‐HA Western blot to verify the ubiquitination of Myc‐PARP1. E, Transfection of Flag‐Smurf2, Myc‐PARP1 and HA‐UB in 293T cells, and the cells were treated with Heclin 25 μmol/L, 2 h, anti‐Myc antibody and Protein A/G immunoprecipitation magnetic beads followed by anti‐Flag by Western blot

Given that Smurf2 can mediate the degradation of PARP1 and that protein degradation is mostly polyubiquitination‐mediated, we transfected cells with empty vector or Flag‐Smurf2 and administered MG132 and Heclin; MG132 and Heclin restored the expression of PARP1, which was consistent with previous results (Figure 6B), suggesting that the degradation of PARP1 is associated with the ubiquitination of Smurf2. In addition, the interaction between Smurf2 and PARP1 was enhanced by MG132 (Figure 6A). Therefore, Smurf2 may degrade PARP1 through enzymatic polyubiquitination.

Smurf2, an E3 ubiquitin ligase, most likely utilizes polyubiquitination to target proteins for degradation. We therefore transfected cells with Flag‐Smurf2 and Flag‐Smurf2 (C716G) and used HA‐UB for coimmunoprecipitation experiments. Smurf2 increased the level of ubiquitinated PARP1 (Figure 6C). Similarly, Smurf2 silencing reduced the level of ubiquitinated PARP1 in Smurf2 knockdown cells (Figure 6D). Further, we transfected Flag‐Smurf2 and HA‐UB and gave Smurf2 inhibitors Heclin for coimmunoprecipitation experiments. Heclin inhibited the ubiquitination of PARP1 (Figure 6E). These experimental results largely suggest that Smurf2 degrades PARP1 through a polyubiquitination pathway and provide some evidence and new ideas for future experiments.

## 4 DISCUSSION

Through the above experiments, we report a newly discovered substrate of the E3 ubiquitinated ligase Smurf2 in the HECT family, PARP1, and reveal that Smurf2 regulates the ubiquitination of PARP1 and mediates the degradation of PARP1. Our results illustrate a new function of Smurf2 and provide insight into the mechanisms of this new Smurf2 function. We also report the role of Smurf2 in oxidative stress‐induced HUVEC injury for the first time.

Smurf2 belongs to the HECT family of E3 ubiquitinated ligases and was originally thought to play a crucial role in embryogenesis, adult tissue homeostasis and the pathogenesis of various human diseases.^19^ As E3 ubiquitination ligase Smurf2 has many binding substrates, such as TGF‐β receptor to regulate TGF‐β pathway,^16^ binding with PTEN to sequential recruitment of activated Akt and NF‐κB‐inducing kinase (NIK) 20 and interacting with DNA topoisomerase IIα (Topo IIα) ensure genomic integrity and unaltered chromosome inheritance.^21^ However, there are few reports of the involvement of Smurf2 in cardiovascular diseases. Our study is the first to find that Smurf2 can participate in cardiovascular system‐related diseases and up‐regulate the expression of proteins related to H_2_O_2_‐induced HUVEC oxidative stress injury. This up‐regulation acts as a protective mechanism for the cell itself. In addition, we confirmed that Smurf2 can interact with PAPR1 by coimmunoprecipitation experiments. Interestingly, one of the functions of Smurf2 as an E3 ubiquitination ligase is to specifically recognize and modify a substrate to determine the fate of the substrate.^22^ At the same time, we found that the expression of Smurf2 can negatively regulate the expression of PARP1. In other words, Smurf2 can affect the stability of PARP1 to a certain extent. These results prove that Smurf2 acts as an E3 ubiquitin ligase and mediates the degradation of PARP1. We also confirmed this finding by Heclin, CHX and MG132 treatment; Smurf2 can degrade PARP1 through the proteasome pathway. Furthermore, we confirmed that PARP1 can be modified by Smurf2‐dependent polyubiquitination and mutated Smurf2 (C716G) or treatment with Smurf2 inhibitor (Heclin) lost its ability to regulate PARP1 expression, further suggesting that Smurf2 promotes the polyubiquitination of PARP1 and then mediates the degradation of PARP1.

PARP1 is involved in many important biological processes and plays important roles in many diseases including inflammation^23^ and tumours.^24^, ^25^ PARP1 is closely related to DNA damage repair under stress conditions.^26^ PARP1 is cut by caspase3 in apoptosis,^11^ which eliminates the enzymatic activity of PARP1, thus completing the biological process of apoptosis.^27^ The stability of single‐ or double‐stranded DNA is reduced or broken in conditions of oxidative stress damage, which activates PARP1. DNA damage caused by ROS and nitrogen species (RNS) is the classical pathway of PARP1 activation.^28^ In H_2_O_2_‐induced cellular oxidative stress damage, cellular DNA breaks, and DNA fragments bind to PARP1 to form a ribosyl‐ribosyl linkage, acting as a signal for other enzymes involved in DNA repair and DNA base repair.^11^ However, PARP1 overactivation results in the depletion of NAD + stored in the cell, which slows glycolysis and electron transfer rates, ultimately affecting the formation of ATP in the cell.^29^, ^30^, ^31^ In addition, NAD + depletion can block glyceraldehyde‐3‐phosphate dehydrogenase activity and affect glycolysis. The cells further deplete intracellular ATP during the synthesis of new NAD+, causing damage to the cells and ultimately leading to cell death.^32^, ^33^ We found that under H_2_O_2_ stimulation, the accumulation of high levels of ROS caused the excessive activation of PARP1, further depleting cellular energy, which led to the apoptosis of HUVECs and the enhanced interaction between Smurf2 and PARP1 under oxidative stress. Smurf2 functions as an E3 ubiquitin ligase that degrades PARP1 via the proteasome pathway and before PARP1 overactivation, thereby effectively preventing PARP1 overactivation under oxidative stress and inhibiting cell apoptosis. Smurf2 thus inhibits cell apoptosis and plays a protective role in alleviating HUVEC injury induced by H_2_O_2_.

We first demonstrated that Smurf2 protects against oxidative stress‐induced HUVEC injury. We confirmed that PAPR1 is a newly described substrate of Smurf2 and that Smurf2 can bind to PARP1. We also confirmed for the first time that Smurf2 regulates the stability of PAPR1 through the proteasome pathway. We acknowledge that our research has certain limitations. We need to confirm the PARP1 ubiquitination site and complete various UB mutations in vivo. We will also perform future experiments in Smurf2 knockout mice to explore the role of Smurf2 in cardiovascular diseases. Our research provides a new therapeutic target for the treatment of cardiovascular diseases, and Smurf2 targeting may be a new method of action in response to vascular endothelial cell injury.

## CONFLICT OF INTEREST

The authors declare that they have no conflicts of interest with the contents of this article.

## AUTHOR CONTRIBUTIONS

YS guided this study. HQ conducted the cells and mechanism part of the experiments. YZ and NZ designed the experiments. BW, SW and SY contributed in the cells and mechanism part of the experiments. HQ and NZ wrote the paper.

## Data Availability

The data used to support the findings of this study are included within the article.
